# Evaluation of the ISO Standard 11063 DNA Extraction Procedure for Assessing Soil Microbial Abundance and Community Structure

**DOI:** 10.1371/journal.pone.0044279

**Published:** 2012-09-11

**Authors:** Pierre Plassart, Sébastien Terrat, Bruce Thomson, Robert Griffiths, Samuel Dequiedt, Mélanie Lelievre, Tiffanie Regnier, Virginie Nowak, Mark Bailey, Philippe Lemanceau, Antonio Bispo, Abad Chabbi, Pierre-Alain Maron, Christophe Mougel, Lionel Ranjard

**Affiliations:** 1 INRA, UMR1347 Agroécologie, Dijon, France; 2 Plateforme GenoSol, INRA, UMR1347 Agroécologie, Dijon, France; 3 Centre for Ecology & Hydrology, Wallingford, United Kingdom; 4 ADEME, Service Agriculture et Forêt, Angers, France; 5 INRA-UEFE, Les Verrines, Lusignan, France; Dowling College, United States of America

## Abstract

Soil DNA extraction has become a critical step in describing microbial biodiversity. Historically, ascertaining overarching microbial ecological theories has been hindered as independent studies have used numerous custom and commercial DNA extraction procedures. For that reason, a standardized soil DNA extraction method (ISO-11063) was previously published. However, although this ISO method is suited for molecular tools such as quantitative PCR and community fingerprinting techniques, it has only been optimized for examining soil bacteria. Therefore, the aim of this study was to assess an appropriate soil DNA extraction procedure for examining bacterial, archaeal and fungal diversity in soils of contrasting land-use and physico-chemical properties. Three different procedures were tested: the ISO-11063 standard; a custom procedure (GnS-GII); and a modified ISO procedure (ISOm) which includes a different mechanical lysis step (a FastPrep ®-24 lysis step instead of the recommended bead-beating). The efficacy of each method was first assessed by estimating microbial biomass through total DNA quantification. Then, the abundances and community structure of bacteria, archaea and fungi were determined using real-time PCR and terminal restriction fragment length polymorphism approaches. Results showed that DNA yield was improved with the GnS-GII and ISOm procedures, and fungal community patterns were found to be strongly dependent on the extraction method. The main methodological factor responsible for differences between extraction procedure efficiencies was found to be the soil homogenization step. For integrative studies which aim to examine bacteria, archaea and fungi simultaneously, the ISOm procedure results in higher DNA recovery and better represents microbial communities.

## Introduction

Soils are considered as complex environments, and are one of the major reservoirs of biological diversity on our planet [1], [2]. Microorganisms (particularly bacteria, archaea and fungi) comprise a significant portion of this huge biodiversity [3], [4]. Recent mathematical computations estimate that one gram of soil can contain between 100,000 and 1,000,000 different bacterial and archaeal species [5]–[7], and although estimates of fungal diversity significantly differ, their species numbers are thought to be in the order of hundreds of thousands to millions [8], [9]. In addition to enormous taxonomic diversity, technical difficulties play a part in the limited understanding of soil microbes. Traditionally, characterization of microbial community composition was limited to microorganisms which could be cultured from environmental samples [1]. It is now known, however, that only a small fraction of microorganisms (less than 1% based on current estimates) are cultivable and therefore accessible for detailed examinations [10], [11]. Over the last three decades, the introduction of culture-independent techniques, based on analyses of microbial DNA, have revolutionized environmental microbiology, yielding a wealth of new information on uncultured microbial populations [1], [12], [13]. As a result, DNA-based phylogenetics of microorganisms is ever-changing and has replaced traditional taxonomy based on morphological, physiological and biochemical parameters [10], [14].

In this context, significant efforts have been devoted to optimize soil DNA extraction procedures to obtain representative extracts for quantitative and qualitative characterization of microbial communities [15]–[18]. This has led to the development of numerous custom DNA extraction protocols as well as commercial kits, each with its own advantages and potential biases [19], [20]. Therefore, different methods should be tested to determine their effects upon soil microbial assessments and to develop easy-to-use, standardized protocols. Based on the method of Martin-Laurent et al. (2001) a standardized “ISO-11063 Soil quality method” was previously developed to directly extract DNA from soil samples [21], [22]. There are several drivers justifying attempts to standardize DNA extraction procedures for the analyses of soil microorganisms, the most notable being to ensure that data are comparable between laboratories to facilitating wider meta-analyses and synthesis. Additionally, standardized procedures are required to provide evidence to policymakers, and many ISO standard methodologies are already available for assessing the biodiversity of larger organisms (see ec.europa.eu/environment/soil/pdf/biodiversity_report.pdf for details). The efficacy and reproducibility of the current ISO DNA extraction method was recently validated by 13 independent European laboratories by comparing the amount of DNA extracted, and the abundance and structure of the bacterial communities in twelve soils [19]. However, as archaea and fungi are also abundant in soil, and are vital functionally, a further evaluation of the ISO-11063 method and other nucleic acids extraction protocols is needed to identify a suitable technique to simultaneously examine these three main groups of soil microbes.

To this end, we compared three different extraction methods: the ISO-11063 [19], [22] (hereon referred to as ISO); the ISOm (a version of the ISO-11063 method modified to include a FastPrep ®-24 mechanical lysis step instead of the recommended beat-beating step using a mini bead-beater cell disruptor); and the GnS-GII, developed by the GenoSol platform to extract soil DNA in large-scale soil surveys (also including a FastPrep ®-24 mechanical lysis step) [20], [23]–[25]. Commercial DNA extraction kits (e.g. Ultraclean soil DNA kit and PowerSoil DNA Isolation Kit from MOBIO, or FastDNA SPIN Kit for Soil from Qbiogene) were not tested in this study, as they have already been evaluated in previous studies with various environments such as soils [12], [20], [21], [26], [27], or activated sludges [28], and have been shown to be less efficient in terms of DNA yield, PCR performance, and/or bacterial diversity estimated by 16S rDNA pyrosequencing. These three soil DNA extraction procedures were used to extract DNA from five contrasting soil types, based on physico-chemical and land-use characteristics. The efficacy of each method was assessed based on estimated microbial biomass (DNA yield), abundance of bacteria, archaea and fungi (semi-quantitative PCR of ribosomal genes), and microbial community structure (terminal restriction fragment length polymorphism (*t*-RFLP) analysis).

## Materials and Methods

### Soil Samples

Five different soils representing forest, grassland and arable biomes were collected from across France (Table 1). All necessary permits were obtained from the respective land owners (INRA, ADEME, and private owners) for the described field studies. Five individual cores (20 cm depth) were sampled at each site using an unaligned sampling design within a defined area. Replicate soil cores were then bulked to obtain a composite sample for each site. After sieving soil samples to <4 mm, aliquots of 50 g were stored at −40°C prior to DNA extraction. Several physico-chemical parameters were measured for each soil: texture, pH, CaCO_3_, and total C and N. Physical and chemical analyses were performed by the Soil Analysis Laboratory of INRA (Arras, France, http://www.lille.inra.fr/las) using standard procedures (Table 1).

**Table 1:** Origins, chemical and physical parameters of the five french soils used.

Soil	Collection site	Origin	Clay	Fineloam	Coarseloam	Finesand	Coarsesand	OrganicCarbon	Total N	C/N	CaCO_3_	pH
C	Agricultural Site	Crop soil	504	180	145	73	98	24.9	2.8	9	102	7.75
E	INRA Experimental Site	Crop soil	392	320	228	34	26	16.5	1.65	10	2	7
F	Forest Observatory Plot	Forest soil	101	167	205	217	310	103.3	3.1	34	<1	3.8
L	INRA Experimental Site ACBB Lusignan	Grassland	175	369	304	73	79	13.2	1.33	9.92	<1	6.6
R	INRA Experimental Site	Crop soil	79	66	44	315	496	50.2	2.16	23.3	22	7.5

Clay, fine loam, coarse loam, fine sand and coarse sand, organic carbon, total N and calcium carbonate are given in mg.g^−1^.

### DNA Extraction Procedures

The three different protocols were adapted to extract DNA from 1 g of soil (dry weight) in order to limit the influence of sampling size on the results obtained for microbial abundance and diversity. DNA was extracted from three technical replicates for each soil sample. All methods were comprised of the same main steps: (a) microbial cell lysis by chemical and physical action; (b) deproteination; and (c) alcohol precipitation and washing of extracted nucleic acids.

#### ISO

This procedure is a modified version of the method described by Martin-Laurent et al. (2001). Soil was added to a bead beating tube containing 0.5 g of glass beads of 106 µm diameter and two glass beads of 2 mm diameter. Each soil sample was first mixed with a solution of 100 mM Tris (pH 8.0), 100 mM EDTA (pH 8.0), 100 mM NaCl, 2% (w/v) polyvinylpyrrolidone (40 g mol^−1^) and 2% (w/v) sodium dodecyl sulfate. Tubes were then shaken for 30 s at 1600 rpm in a mini bead-beater cell disruptor (Mikro-Dismembrator; S.B. Braun Biotech International) before centrifugation at 14,000×g for 1 min. After removing the supernatant, proteins were precipitated, with 1/10 volume of 3 M sodium acetate prior to centrifugation (14,000×g for 5 min at 4°C). Finally, nucleic acids were precipitated by adding 1 volume of ice-cold isopropanol. The DNA pellets obtained after centrifugation (14,000×g for 5 min at 4°C) were washed with 70% ethanol (full details are described in [21], [22]).

#### GnS-GII

This DNA extraction procedure was developed at the platform GenoSol (http://www.dijon.inra.fr/plateforme_genosol) for large-scale soil surveys and has recently been compared to other protocols [20]. Briefly, in a 15 ml Falcon tube each soil sample was mixed with 4 ml of a solution containing 100 mM Tris (pH 8.0), 100 mM EDTA (pH 8.0), 100 mM NaCl, and 2% (wt/vol) sodium dodecyl sulphate. Two g of 100 µm diameter silica beads, 2.5 g of 1.4 mm diameter ceramic beads and 4 glass beads of 4 mm diameter were added to the mixture. Samples were then homogenized for 3×30 s at 4 m.sec^−1^ in a FastPrep ®-24 (MP-Biomedicals, NY, USA). The samples were incubated for 30 min at 70°C, then centrifuged at 7,000 × g for 5 min at 20°C. To remove proteins from the extracts, 1 ml of supernatant was incubated for 10 min on ice with 1/10 volume of 3 M potassium acetate (pH 5.5) then centrifuged at 14,000×g for 5 min. Finally, after precipitation with one volume of ice-cold isopropanol, nucleic acids were washed with 70% ethanol.

#### ISOm

This composite procedure is the same as the ISO procedure, except for the lysis step. Briefly, this particular step was done by mixing each soil sample with a solution of 100 mM Tris (pH 8.0), 100 mM EDTA (pH 8.0), 100 mM NaCl, 2% (w/v) polyvinylpyrrolidone (40 g mol^−1^), and 2% (w/v) sodium dodecyl sulfate. Then, 2 g of 100 µm diameter silica beads, 2.5 g of 1.4 mm diameter ceramic beads and 4 glass beads of 4 mm diameter were added to the mixture. The samples were then homogenized for 3×30 s at 4m sec^−1^ in a FastPrep ®-24 (MP-Biomedicals, NY, USA). The samples were finally incubated for 30 min at 70°C, and then centrifuged at 7,000×g for 5 min at 20°C. Subsequent steps were then performed as described above for the ISO protocol.

### Crude Soil DNA Quantification

Crude DNA extracts for all DNA extraction procedures were resolved by electrophoresis in a 0.8% agarose gel, stained with ethidium bromide and a picture of each gel was acquired (Infinity-Capt, Vilber Lourmat, Marne-la-Vallée, France). Dilutions of calf thymus DNA (BIORAD, Marne-la-Coquette, France) were included in each gel and a standard curve of DNA concentration (31.25 to 500 ng) was used to estimate the final DNA concentration in the crude extracts [29]. The ethidium bromide fluorescence intensity was integrated with ImageQuaNT software (Molecular Dynamics, Evry, France). The reliability of this method in limiting bias due to soil impurities that can hamper DNA quantification has been confirmed [29].

### Purification and Quantification of Soil DNA Extracts

As the DNA purification step is not part of the ISO protocol, all crude soil DNA extracts were purified using the same procedure [29]. Briefly, nucleic acids were separated from the residual impurities, particularly humic substances, by centrifuging through two types of minicolumn. Aliquots (100 µl) of crude DNA extract were first loaded onto PVPP (polyvinylpolypyrrolidone) minicolumns (BIORAD, Marne-la-Coquette, France) and centrifuged at 1,000×g for 2 min at 10°C. The eluate was then purified using the Geneclean turbo kit (Q-Biogene, Illkirch, France). Purified DNA concentrations were finally assessed using the PicoGreen (Molecular Probes, Paris, France) staining kit, according to the manufacturer’s instructions.

### Semi-quantitative PCR Assays

Bacterial, fungal and archaeal semi-quantitative PCR assays, were performed using an ABI PRISM 7900HT (Applied Biosystems, Courtaboeuf, France) with a SYBRGreen® detection system. DNA was amplified in a total reaction volume of 20 µl, containing 500 ng of T4 gene 32 protein (MP Biomedicals, France) and 10 µl of SYBR Green PCR master mix (Applied Biosystems, France).

For bacterial quantification, the reaction mixtures contained 1 µM of each primer (341F: 5′ - CCTACGGGAGGCAGCAG - 3′ and 515R: 5′ - ATTACCGCGGCTGCTGGCA - 3′) [30], and 1 ng of template DNA. The PCR conditions consisted of an initial step of 15 min at 95°C then 35 cycles of 15 s at 95°C, 30 s at 60°C, 30 s at 72°C and 20 s at 80°C. The 16S rRNA gene from a pure culture of *Pseudomonas aeruginosa* PAO (INRA Dijon collection) was used as standard for the bacterial semi-quantitative PCR assay.

Soil fungi were quantified using 1.25 µM of each primer (FR1: 5′-AICCATTCAATCGGTAIT-3′, and FF390: 5′-CGATAACGAACGAGACCT-3′) [23], and 2.5 ng of template DNA. The PCR conditions were: an initial step of 10 min at 95°C for activation; followed by 40 cycles of 15 s at 95°C, 30 s at 50°C and 60 s at 70°C. Amplified DNA from a pure culture of *Fusarium oxysporum* 47 (INRA Dijon fungal collection) was used as a fungal standard.

To quantify soil archaea, 10µM of each primer were used (771F: 5′-ACGGTGAGGGATGAAAGCT-3′, and 957R 5′-CGGCGTTGACTCCAATTG-3′) [31], with 2 ng of template DNA. The amplification conditions were: an initial step of 15 min at 95°C followed by 35 cycles of 15 s at 95°C, 30 s at 55°C, 30 s at 72°C and 30 s at 80°C.

For the three different semi-quantitative PCR protocols, a final temperature step from 60°C to 95°C of 0.5°C sec^−1^ increments was added to obtain a specific denaturation curve. Purity of the amplified products was checked by observation of a single melting peak.

### DNA Fingerprinting Method: t-RFLP

To examine soil bacterial, fungal and archaeal communities extracted using the different methods, *t*-RFLP analyses were performed. All PCR reactions took place in a volume of 50 µl. Bacterial 16S rRNA genes were amplified using forward primer 63F (5'-CAGGCCTAACACATGCAAGTC-3') [32] labeled at the 5′ end with 6-FAM fluorescent dye, and reverse primer 519r (5′-GTATTACCGCGGCTGCTG-3′) [33]. Amplifications were carried out under the following conditions: 94°C for 1 min 30 s; then 35 cycles of 94°C for 45 s, 55°C for 60 s and 72°C for 1 min 30 s; followed by a single step of 72°C for 5 min. Fungal communities were analyzed using primers 6-FAM labeled ITS1F (5′-CTTGGTCATTTAGAGGAAGTAA-3′) and ITS 4 (5′-TCCTCCGCTTATTGATATGC-3′) [34] using the following conditions: 95°C for 4 min; followed by 35 cycles of 94°C for 45 s, 53°C for 60 s and 72°C for 1 min 30 s; then a final elongation of 72°C for 5 min. The archaeal assay was carried out with the primer pair 6-FAM labeled A364aF (5′-CGGGGYGCASCAGGCGCGAA-3′) [35] and A934b (5′-GTGCTCCCCCGCCAATTCCT-3′) [36] using the following conditions: 94°C for 4 min; followed by 30 cycles of 94°C for 45 s, 52°C for 60 s and 72°C for 60 s; and a final elongation step of 72°C for 10 min.

Following amplification, fluorescently labeled amplicons were purified by gel filtration with Sephadex G50 (Sigma-Aldrich, Gillingham, UK) by spinning at 450×g for 5 min at 4°C. Purified PCR product (50 ng) was digested with restriction enzyme *Msp* 1 (New England Biolabs Inc., Ipswich, MA, USA) for bacteria, and *Taq* 1 (New England Biolabs Inc., Ipswich, MA, USA) for fungi and archaea, according to the manufacturer’s guidelines. Restriction digests were mixed with Hi-Di formamide and GeneScan–600 LIZ size standard (Applied Biosystems, Cheshire, UK), and fragment analysis was done using a 3730 DNA analyser (Applied Biosystems, Cheshire, UK). Resulting data were analysed by peak height analysis using the binning option within the GeneMarker software (SoftGenetics, PA, USA). Relative abundance of amplicons was estimated as the ratio between the integrated fluorescence of each of the T-RFs and the total integrated fluorescence of all the T-RFs.

### Statistical Analyses

A Mann – Whitney test was used to analyze the effects of the DNA extraction procedure on the amounts of extracted DNA and ribosomal gene copy number for bacterial, fungal and archaeal communities. Significance was assessed at the level of *p*<0.05.

To examine the effects of extraction method and soil type on microbial community structure, multivariate analyses of *t*-RFLP data were carried out with the Vegan package http://cc.oulu.fi/~jarioksa/softhelp/vegan.html) in R. Permutational multivariate analysis of variance (perMANOVA) was performed using the adonis function, and principal components analysis (PCA) of *t*-RFLP data was carried out using the rda function from R [37].

## Results and Discussion

Bacteria, archaea and fungi are of particular importance for ecosystem functioning as they are integral to soil processes, such as organic matter transformation, and nutrient and biogeochemical cycling [38], [39]. A variety of DNA extraction procedures have been developed to monitor soil microbial communities; however, the large number of molecular methods employed makes it difficult to compare results obtained across different studies. The generic use of the same soil DNA extraction method between studies will improve data comparison, increasing our knowledge and synthesis of the factors determining soil microbial diversity. Therefore the ideal DNA extraction procedure has to be suitable for use on a wide range of soils, and allow the studying of bacterial, archaeal and fungal communities from the same DNA extract.

### Influence of DNA Extraction Procedure on Crude Soil DNA Yield

**Figure 1:**
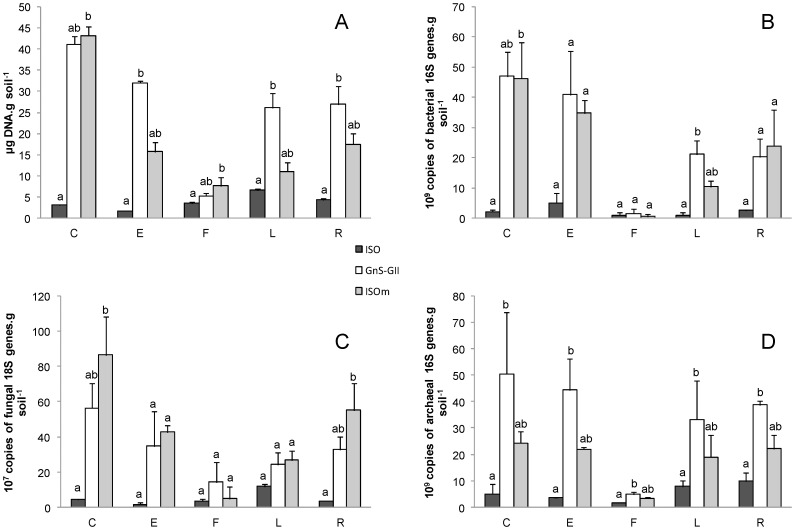
Quantifications of crude extracted DNA and microbial abundances according to extraction procedures in different soils. Quantification of (A) crude extracted DNA, (B) 16S rRNA genes, (C) 18S rRNA genes, (D) archaeal 16S rRNA genes according to three different extraction procedures (ISO, GnS-GII and ISOm) in five different soils (C, E, F, L, R). Bars correspond to averages of three replicates ± SD (n = 3). Within each soil, bars topped by the same letter are not significantly different at *p*<0.05.

Crude DNA was successfully extracted from all soils using each of the three different DNA extraction methods (Figure 1A). It is important to note that an increase in DNA yield was not associated with greater shearing of DNA (as visualized by gel electrophoresis, data not shown). For all three methods the amounts of DNA recovered after purification (varying from 1 to 20 µg g soil^−1^) are of the same magnitude as previously reported [17], [19], [40]–[42]. DNA yield is strongly dependent upon soil type, pH, organic matter, clay and silt content as these factors can influence either the growth of certain microbial taxa, or the formation of aggregates which host microorganisms [17], [43]–[45]. However, DNA yield is not the only indicator of DNA extraction efficacy. Indeed, greater amounts of DNA do not necessarily mean that a greater number of taxa can be detected. It is likely that extracted DNA mainly comes from easily lyzed cells and easily lyzed aggregates [17], [46], and therefore, differences in microbial cell wall structure and microhabitats will affect the extraction of DNA and thus the analyses of diversity.

Overall, the ISO procedure yielded significantly less DNA (mean = 3.87±0.23 µg DNA g^−1^ soil), than the ISOm (mean = 19.03±2.22 µg DNA g^−1^ soil), and the GnS-GII (mean = 26.26±2.20 µg DNA g^−1^ soil) procedures (Figure 1A). The higher efficiencies of GnS-GII and ISOm may be a result of the common mechanical lysis step in these protocols. The FastPrep ®-24 bead beating system is thought to break open more cells, compared to usual bead-beating [12], [20], [44].Whilst stronger or longer physical treatments may improve microbial cell breakdown resulting in higher DNA yields, they may also cause significant shearing of DNA [47], [48]. Soil type also had an effect on DNA yield. Interestingly, the greatest amount of DNA was extracted from an arable, calcareous soil (soil C), whereas the smallest amount was detected in an acidic, sandy forest soil with high organic carbon content and high C:N ratio (soil F). These differences were clearly shown with the GnS-GII and the ISOm procedures (DNA yield was much lower in the acidic soil under beech and coniferous forest), but not with the ISO method. Our results with the GnS-GII and the ISOm protocols confirm the impact of soil pH and land-use on soil microbial biomass, as already demonstrated in previous studies [47], [49]. Consequently, these results highlight the need to use a DNA extraction protocol with enough sensitivity to detect quantitative changes between soils of differing characteristics and management. This is particularly the case when the amount of soil DNA is to be used as an indicator of soil microbial biomass [23], [50]–[53].

### Influence of Soil DNA Extraction Procedure on Bacterial, Archaeal and Fungal Densities

A semi-quantitative PCR approach was used to compare the abundances of different microbial groups (bacteria, archaea and fungi) in various French soils using DNA which had been extracted with three different methods. Although semi-quantitative PCR targeting ribosomal RNA gene sequences does not provide an absolute measure of biomass, because of gene copy number fluctuations in bacterial and archaeal taxa, and because the number of nuclei per cell varies amongst fungal species, it can still give a good metric to track shifts in the relative abundance of bacteria, fungi and archaea [47], [54]–[57].

The detected total eubacterial 16S rRNA gene copy numbers per g of soil ranged from 0.58×10^9^ in soil F with the ISOm procedure to 46.94×10^9^ in soil C using the GnS-GII and ISOm procedures (Figure 1B). Significant differences in relative 16S rRNA gene copy numbers were measured between the GnS-GII and ISO protocols for C and L soils. Indeed, when looking at the average quantifications, the GnS-GII and ISOm methods detected 11 and 10 times more 16S rRNA gene copies than the ISO method respectively. Moreover, with these two protocols, significant differences in total 16S rRNA copies between C and F soils were found, whereas no significant difference was observed with the ISO protocol between these two soils.

For fungal rRNA gene quantification, the largest and smallest copy number per g of soil were measured in soils C (86.35×10^7^) and E (1.50×10^7^) with the ISOm and ISO procedures respectively (Figure 1C). For the C and R soils 18S rRNA gene copy number was significantly different between the ISOm and the ISO protocols. Moreover, in the five soils, the ISOm and GnS-GII procedures were more efficient at detecting fungal communities. Indeed, they respectively recovered an average of 8.7 and 6.5 times more 18S rRNA gene copies per g of soil than the ISO protocol. Meanwhile, fungal abundance was only significantly different between soils using the ISO and ISOm procedures. Similar to the results obtained for DNA yield, 18S rRNA gene copy number was different between L and E soils with ISO-extracted DNA, and C and F soils using ISOm DNA extracts.

Archaeal 16S rRNA genes were detected from the five soils, using all extraction procedures. Abundances ranged from 1.77×10^9^ (F soil, ISO extraction) to 50.44×10^9^ (C soil, GnS-GII extraction) copies per g of soil (Figure 1D). For all soils, archaeal abundances were significantly different between the ISO and GnS-GII procedures. Moreover, increased archaeal abundances were measured with the ISOm method compared to the ISO, however this was not statistically significant. Based on these results, the ISOm and GnS-GII procedures revealed higher archaeal abundances, as these two methods respectively detected 3.2 and 6.1 times more 16S rRNA gene copies than the ISO protocol. Lastly, the abundance of archaea was similar between the remaining soils, with the exception of the F soil which had less archaeal 16S rRNA genes. For the ISO DNA extracts only, a significant difference between archaeal 16S rRNA gene copy number between soils F and R was found.

Although only a limited number of soils were used in the present study, it is interesting to note that the detected bacterial abundances were significantly lower in soil F which is of a low pH (Table 1 and Figure 1B). As already known, soil pH has a strong impact on the abundance of bacterial communities, and our findings corroborate the positive relationship between bacterial abundance and soil pH (e.g. [23]). Furthermore, our results also showed that the fine-textured soil (C) exhibited a higher fungal abundance than the coarse-textured soil (F), only with the ISOm DNA extraction procedure. This is in accordance with a recent study which has also found that fungal abundance, estimated by semi-quantitative PCR on 24 independent soils of contrasting physico-chemical characteristics and land-use type, was significantly correlated with soil physico-chemical properties (texture, C_org_ content and C:N ratio), but not clearly with other soil parameters (e.g. soil pH) [22].

As already demonstrated in previous studies, the results obtained for these three taxonomic groups studied suggest that assessments of soil microbial abundance can be skewed according to the procedures used to recover DNA from soil. For example, significant differences of detected 16S and 18S gene copies between C and F soils were found using the ISOm protocol, but not the ISO and the GnS-GII methods. Therefore, we can conclude from these results that the ISOm and the GnS-GII procedures are more efficient at extracting bacterial, archaeal and fungal DNA from different types of soils, indicating that the FastPrep ®-24 bead beating system breaks open more cells than the beat-beating step defined in the ISO method. This is somewhat unsurprising as the ISO standard was originally designed to study bacterial communities [18], [58]–[60].

### Influence of DNA Extraction Procedure on Microbial Community Structure

The structure of bacterial, fungal and archaeal communities in the five studied soils was analyzed by *t*-RFLP. This technique, shown to be well adapted for analyzing a large number of samples and for detecting differences in the diversity and composition of bacterial communities [59], gives a fingerprint for the three microbial domains, based on the length and abundance of unique restriction fragments in each sample. The main drawback of this method is that it gives an underestimated representation of microbial diversity, as only a limited number of terminal restriction fragments can be detected for each sample, and often a single terminal restriction fragment can be shared by several species [18], [47], [54]–[57].

**Figure 2:**
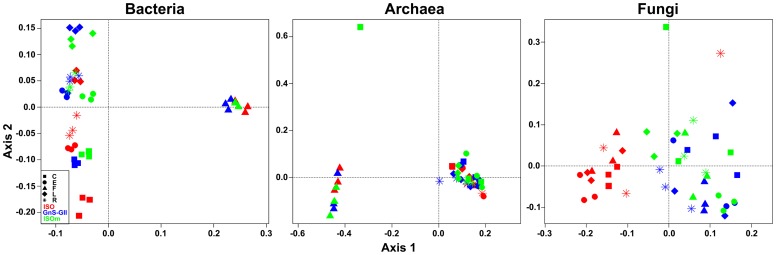
Principal component analysis of microbial communities t-RFLP profiles according to DNA extraction procedures. Principal component analysis of t-RFLP profiles of (A) bacterial communities, (B) archaeal communities, and (C) fungal communities, coming from five different soils (C, E, F, L, R) according to three different extraction procedures (ISO, GnS-GII and ISOm).

When examining the PCA results (Figure 2A), clear separation of bacterial communities based on soil type was observed. In particular, forest soil F separated from the other soils along the first axis. This soil differs in a number of physicochemical variables compared to the other samples (Table 1). The remaining soil samples were distributed vertically across the second axis, with the C (pH 7.75) and L (pH 6.6) soils shown to be the most different. Slight variations in extraction method were also observed as samples clustered closely together according to the ISO, GnS-GII or ISOm procedure used. These results are in agreement with many other studies which have reported upon the relationship between soil bacterial community structure and soil pH (e.g. [23], [39], [57]). We then examined the relative influence of soil type and extraction method in explaining the variance in bacterial communities, using perMANOVA tests (Table 2), concluding that, for these samples, any of the extraction methods can provide a representative picture of the community and reveal the effects of different soil types in predicting community structure.

**Table 2:** PerMANOVA analyses of microbial communities t-RFLP profiles : influence of extraction method and soil type.

	Bacteria		Archaea		Fungi	
	*F*	*R^2^*	*F*	*R^2^*	*F*	*R^2^*
Extraction method	11.23	0.03*	0.76	0.006	27.29	0.34*
Soil type	108.63	0.78*	54.50	0.84*	11.33	0.29*
Interaction	4.72	0.08*	1.09	0.03	3.62	0.18*

PerMANOVA analysis showing the influence of extraction method and soil type in explaining overall variance in microbial communities.

* denotes significance (*p*<0.01).

Similarly, the archaeal communities in soil F were distinct from other soils along the primary axis, regardless of extraction method (Figure 2B). This agrees with previous studies showing that soil pH was a factor driving archaeal diversity [59]. However, within the remaining samples there were no clear extraction method or soil type differences, highlighting the need for a more thorough examination of the potential link between archaeal community composition and other soil physico-chemical parameters. Finally, the perMANOVA (Table 2) confirmed that the three extraction methods provide a representative discrimination between archaeal communities from different soil types.

Fungal diversity patterns were mainly affected by the DNA extraction procedure (Figure 2C) as almost all samples analyzed using the ISO method grouped away from the other samples along the first axis. When all samples were examined simultaneously there appeared to be no separation of fungal communities by soil type. Moreover, based on perMANOVA results (Table 2), soil fungal community structure was less well predicted by soil type, and the choice of extraction method explained a larger proportion of the variance in fungal communities. However, separate examination of fungal communities extracted using the different methods showed that, with the GnS-GII and ISOm methods, fungal communities in the low pH soil were clearly different (see Figure S1), corroborating other studies in which soil characteristics were shown to impact upon fungal community structure [10], [12], [15], [20].

We then sought to examine which of the three extraction methods were the most effective at discriminating soil type differences in bacterial, archaeal and fungal communities (Table 3). As inferred previously, for bacteria and archaea all methods used were very effective in discriminating community differences due to soil type (*R^2^*>0.79). However for fungi, the two non-ISO methods clearly outperformed the ISO method in being able to detect variation in community structure due to soil type. These differences between extraction methods are thought to be due to a less efficient soil mechanical lysis in the ISO procedure. The main difference between the ISO and the two other procedures is the homogenization step. Compared to traditional bead-beating the FastPrep ®-24 bead beating system is thought to lyze the majority of cells with tough walls, especially fungal cells [24], [39], [47], [57], [61]. Similar results have been found for soil microbial communities using automated ribosomal intergenic spacer analysis (ARISA) (data not shown).

**Table 3:** PerMANOVA analyses of microbial communities t-RFLP profiles : effect of soil type.

	Bacteria		Archaea		Fungi	
	*F*	*R^2^*	*F*	*R^2^*	*F*	*R^2^*
ISO	56.39	0.96*	50.62	0.95*	2.09	0.46*
GnS-GII	37.38	0.94*	34.29	0.93*	8.41	0.77*
ISOm	31.34	0.93*	9.48	0.79*	9.29	0.79*

PerMANOVA analysis showing the effect of soil type on microbial communities assessed by the three extraction methods.

* denotes significance (*p*<0.01).

Altogether, the three microbial communities differed between some of the five sites suggesting that environmental factors help to shape unique communities of fungi, bacteria, and archaea. In the case of bacteria, these factors include soil pH, C:N ratio, organic carbon content and texture, as already described in previous studies or reviews [5], [39]. On the other hand, archaeal and fungal diversity patterns differences also arose from variations in soil properties (i.e. pH), but discrepancies between our results and recent studies highlight the need for a more thorough examination of the potential link between archaeal community composition and soil physico-chemical parameters [18], [54], [56], [57], [62]. Lastly, our observations agree with previous work showing that soil physical and chemical characteristics (in particular soil pH) can influence strongly microbial community structure [18], [47]. Here, soil F had a lower pH than the other soils (Table 1) and harbored unique microbial communities compared to the four other soils.

### Conclusion

We have shown that the choice of DNA extraction method can have a significant effect upon bacterial, archaeal and fungal molecular analyses and is therefore an important consideration for microbial studies. This was particularly the case for soil fungi as increased fungal abundances were detected using the ISOm method, and extraction protocol was generally found to have more of an effect upon fungal community structure than soil type. Specifically, the effects upon community structure were less pronounced using the ISO method compared to the other two procedures. However, greater yields of DNA and increased abundances were measured with the GnS-GII and ISOm techniques.

These results have also demonstrated that for a comparative analysis of soils and different microbial groups, a single DNA extraction method must be used. Among the three methods we evaluated, we propose the adoption of the ISOm method to study bacteria, archaea and fungi, as it was a slight modification of the existing ISO-11063 protocol, through a mechanical lysis step using the FastPrep ®-24 (increasing soil DNA yields), instead of the recommended beat-beating. The next step to evaluate this procedure will be the assessment of this method using soils with a wider range of physico-chemical characteristics from large scale surveys (spatial and/or temporal) [1], [20] and soils from particular and extreme environments (e.g. volcanic soils, artic soils, saline soils, etc…) [63]–[65]. Then, an inter-laboratory validation must be made, potentially employing new high throughput sequencing technologies to allow more detailed examination of the differences in community patterns detected as a result of extraction procedure.

## Supporting Information

Figure S1 **Principal component analysis of fungal communities t-RFLP profiles according to DNA extraction procedures.** Principal component analysis of t-RFLP profiles obtained from (A) ISO DNA extraction, (B) GnS-GII DNA extraction, and (C) ISOm DNA extraction, coming from five different soils (C: ▪, E: •, F: ▴, L: ♦, R: *) according to three different extraction procedures (ISO, GnS-GII and ISOm).
(DOC)
