# Supplementary material for: Evaluation of the ISO Standard 11063 DNA Extraction Procedure for Assessing Soil Microbial Abundance and Community Structure
Source: PLoS One. 2012 Sep 11;7(9):e44279. doi: 10.1371/journal.pone.0044279 (PMC3439486; doi:10.1371/journal.pone.0044279)
Supplement: Figure S1 — Principal component analysis of fungal communities t-RFLP profiles according to DNA extraction procedures. Principal component analysis of t-RFLP profiles obtained from (A) ISO DNA extraction, (B) GnS-GII DNA extraction, and (C) ISOm DNA extraction, coming from five different soils (C: ▪, E: •, F: ▴, L: ♦, R: *) according to three different extraction procedures (ISO, GnS-GII and ISOm). (DOC) [file pone.0044279.s001.doc]

|  |  |  |
| --- | --- | --- |

**Figure S1. Principal component analysis of fungal communities t-RFLP profiles according to DNA extraction procedures**. Principal component analysis of t-RFLP profiles obtained from (A) ISO DNA extraction, (B) GnS-GII DNA extraction, and (C) ISOm DNA extraction, coming from five different soils (C: ■, E: ●, F: ▲, L: ♦, R: *) according to three different extraction procedures (ISO, GnS-GII and ISOm).
